# Crosstalk between zinc and free fatty acids in plasma

**DOI:** 10.1016/j.bbalip.2018.09.007

**Published:** 2019-04

**Authors:** James P.C. Coverdale, Siavash Khazaipoul, Swati Arya, Alan J. Stewart, Claudia A. Blindauer

**Affiliations:** a Department of Chemistry, University of Warwick, Coventry CV4 7AL, UK; b School of Medicine, University of St Andrews, St Andrews KY16 9TF, UK

**Keywords:** ACB, albumin‑cobalt binding, AD, Alzheimer's disease, BSA, bovine serum albumin, DTT, dithiothreitol, FA1-7, fatty acid binding sites 1–7, FFAs, free fatty acids, HRG, histidine-rich glycoprotein, HSA, human serum albumin, IMA, ischemia-modified albumin, ITC, isothermal titration calorimetry, mol. equiv., molar equivalents, Myr, myristate, PUFA, poly-unsaturated fatty acid, Zinc, Non-esterified fatty acids, Plasma, Serum, Albumin

## Abstract

In mammalian blood plasma, serum albumin acts as a transport protein for free fatty acids, other lipids and hydrophobic molecules including neurodegenerative peptides, and essential metal ions such as zinc to allow their systemic distribution. Importantly, binding of these chemically extremely diverse entities is not independent, but linked allosterically. One particularly intriguing allosteric link exists between free fatty acid and zinc binding. Albumin thus mediates crosstalk between energy status/metabolism and organismal zinc handling. In recognition of the fact that even small changes in extracellular zinc concentration and speciation modulate the function of many cell types, the albumin-mediated impact of free fatty acid concentration on zinc distribution may be significant for both normal physiological processes including energy metabolism, insulin activity, heparin neutralisation, blood coagulation, and zinc signalling, and a range of disease states, including metabolic syndrome, cardiovascular disease, myocardial ischemia, diabetes, and thrombosis.

## Highlights

• Serum albumin binds and transports both free fatty acids and Zn^2+^ ions
• Elevated plasma free fatty acids impair Zn^2+^ binding by albumin through an allosteric mechanism
• The resulting changes in plasma zinc speciation are thought to impact blood coagulation and may promote thrombosis
• Increased free Zn^2+^ may lead to enhanced zinc export from plasma and dysregulation of zinc homeostasis in multiple tissues

## Graphical abstract

**Figure:**
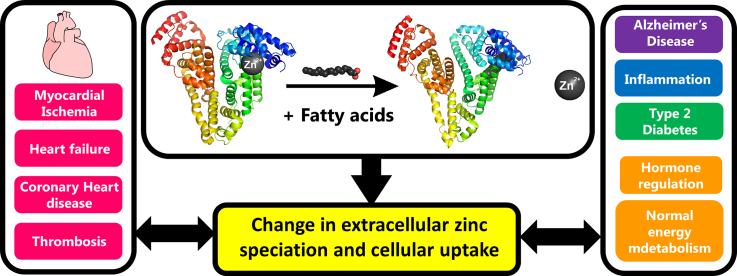

## 1 Introduction

Zinc is the second-most abundant d-block metal in the body (after iron). It is considered essential for life in both eukaryotic and prokaryotic organisms, since it is required for development and function of all cells [[1], [2], [3], [4], [5]]. Zn^2+^ ions are estimated to be present in 10% of all mammalian proteins [6,7], where they can play structural (most notably in zinc finger motifs), catalytic or regulatory roles. Most recently, Zn^2+^ has been recognised as a signalling agent [2,8,9] and plays critical roles in determining the activity and fate of cells [10]. This may also at least partially account for the surprisingly high toxicity of uncomplexed (=free) Zn^2+^ [[11], [12], [13]]. For these reasons, Zn^2+^ homeostasis in all organisms including humans is tightly regulated [14]. Dietary zinc deficiency (hypozincemia) is commonly associated with impaired growth, infertility, and immunodeficiency [2], but an increasing number of genetic and other disorders are characterised by defective zinc transport into particular cells or their sub-compartments [4,15]. A particularly drastic example of genetically encoded zinc dyshomeostasis is the ‘lethal-milk syndrome’ in mice. This arises from a non-sense mutation in the gene for the Zn^2+^ transporter ZnT4, resulting in defective secretion of Zn^2+^ into breast milk [16]. In humans, a Gly-to-Arg mutation in the functionally analogous ZnT2 transporter leads to transient neonatal zinc deficiency [17]. Zinc dyshomeostasis may also be a hallmark of ageing and several neurological disorders [[18], [19], [20]]. Direct and indirect effects of zinc on hormonal signalling are also pervasive [8,21,22]. Furthermore, zinc is known to be crucial for immune system function in multiple ways [[23], [24], [25]]. Apart from the requirement of zinc for normal cellular functions [24], zinc also acts as a signalling agent to modulate (immune) cell activity [25]. Moreover, both zinc sequestration and zinc toxicosis are used as strategies by the human host during bacterial and fungal infections [26].

The diverse biochemistry of zinc means that, in many cases, more than one factor (or pathway) is at play. Whilst catalytic Zn^2+^ sites are now typically well-understood, the recognition that Zn^2+^ levels regulate the function of many cell types has led to vigorous interest in the fate and role of mobile Zn^2+^ in the body. Much attention has focused on membrane-bound zinc transporters that mediate uptake, efflux and sub-cellular compartmentalisation [4], and the development of methodology to measure the concentrations of free intracellular Zn^2+^ [27,28]. Recent work has highlighted a central role for the extracellular medium to determine the effects of Zn^2+^ on cells [13]. Two aspects need to be considered: the total concentration of Zn^2+^ in the medium, and its speciation. Speciation refers to the ‘form’ in which Zn^2+^ ions are present, e.g. bound to a particular protein, other biomolecules, or ‘free’ (i.e. the uncomplexed aquo-ion). This is not only relevant for *in vitro* cell culture, but impacts on whole-organism zinc distribution, as this is orchestrated in the blood plasma. The unexpected powers of “young plasma” to counter-act the deterioration of the ageing brain [29] and serum albumin's therapeutic efficacy in the treatment of Alzheimer's disease [30] have been highlighted recently [31]. We propose that alongside other mechanisms, this important extracellular medium and its most abundant protein take an active role in the dynamic management of whole-body zinc fluxes.

In this review, we examine the interplay between Zn^2+^ homeostasis and fatty acid metabolism. Free (or non-esterified) fatty acids (FFAs) have been strongly implicated in the modulation of plasma zinc speciation, via an allosteric switch on serum albumin [32]. We highlight that a quantitative approach to studying this dynamic system gives access to understanding fluctuations in Zn^2+^ concentrations and speciation, and how a range of physiological processes and disease states may be affected.

## 2 Albumin is the main plasma carrier of zinc and fatty acids

**Fig. 1:**
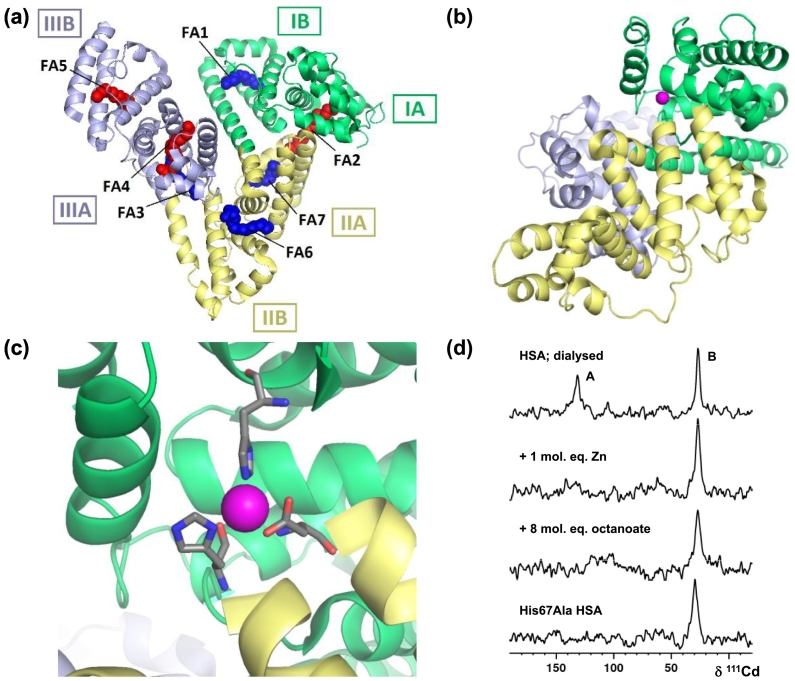
Serum albumin can bind free fatty acids and zinc ions. (a) High-affinity (**red**) and low-affinity (**blue**) FA binding sites 1–7 of the human serum albumin-palmitate complex (PDB: 1E7H) [57]. The roman numerals and letters denote the three domains and each subdomain of albumin. Each homologous domain (labelled I-III) of HSA is divided into two subdomains. Figure adapted from Fujiwara & Amisaki, 2013 [58]. (b) Location of the interdomain zinc (**purple**) binding site A (PDB: 5IJH) [33]. (c) Tetrahedral coordination of His67, His247 and Asp249 to Zn^2+^ in site A (PDB: 5IJH) [33]. The fourth ligand, a water molecule, is not shown. (d) ^111^Cd-NMR has aided in identification of zinc binding site A on human serum albumin. One equivalent of zinc is sufficient to displace Cd from site A. Mutation of His67 shows this amino acid to be essential to Cd^2+^ binding, and addition of fatty acids interferes with metal binding to site A [48].

The majority of mobile Zn^2+^ in plasma binds to serum albumin [33] (Fig. 1a), a 66 kDa protein that constitutes approximately 60% of the total plasma protein content [34]. Approximately 75–90% of plasma zinc is albumin-bound, with the remaining 10–20% bound to either α2-macroglobulin or the retinol-binding protein complex [35,36]; the firmly bound Zn^2+^ in the latter two proteins is not part of the labile pool. Although a role for transferrin in plasma Zn^2+^ binding has been suggested, strong past and present evidence argues against this contention [35,37,38]. Only low nanomolar concentrations of ‘free’ Zn^2+^ are present in blood plasma under normal conditions [39,40]. Serum albumin also reversibly binds a range of small compounds, including drugs and hormones [41], affecting both their bio-distribution and bio-availability [34,42]. Albumin is thought to affect cellular Zn^2+^ uptake in direct and indirect ways. Whilst ‘free’ Zn^2+^ is readily accumulated by endothelial cells, albumin also appears to permit the uptake of Zn^2+^. Both ultra-filtrated (this includes both free Zn^2+^ and low molecular mass zinc complexes) and dialysed (protein-bound Zn^2+^) ^65^Zn-labelled serum fractions contributed to the accumulation of Zn^2+^ in cells [43]. Other studies have suggested that cellular zinc accumulation could involve receptor-mediated endocytosis, with Zn^2+^ co-transported by albumin [44].

The most important metal binding site on albumin is site A (Fig. 1b and c), commonly referred to as a ‘multi-metal’ binding site [45], as it binds a variety of d-block metal ions including Zn^2+^, Cd^2+^, Cu^2+^, Ni^2+^ and Co^2+^ [46]. In agreement with the Irving-Williams series, Cu^2+^ will preferentially coordinate over the other transition metal ions, but only at stoichiometries exceeding 1:1, which does not normally occur *in vivo* [47]. Zn^2+^ binding at site A was initially confirmed by ^111^Cd and ^113^Cd NMR spectroscopy (isotopes with a nuclear spin of *I* = ½, 12.8% and 12.2% natural abundance, respectively) which showed two resonances at 113–124 ppm (site A) and a second site at 24–28 ppm (site B), both of which are indicative of coordination by a mixture of nitrogen/oxygen ligands [36,48,49]. The high affinity of site A for Zn^2+^ was demonstrated by the facile displacement of Cd^2+^ by only one equivalent of Zn^2+^ (Fig. 1d) [49,50]. More recently, both human (HSA) and equine serum albumin (ESA) have been successfully crystallised with bound Zn^2+^, confirming the inferred inter-domain location of site A between domains I and II (Fig. 1c), and revealing the tetrahedral coordination geometry for Zn^2+^ binding, involving His67, His247, Asp249, and a water molecule (Fig. 1c). The affinity of this site for Zn^2+^ [12,[51], [52], [53], [54], [55], [56]] lies in the mid to high nanomolar range, depending on animal species and conditions. Most studies on Zn^2+^ binding to albumins have reported either two or three significant binding sites, with site B thought to correspond to the second-strongest site, with the N-terminus the favourite candidate for the third site [36]. ^111^Cd NMR on BSA [55] and the recent X-ray studies on HSA [33] suggested that site B might also be an inter-domain site.

Albumin is the major carrier of free fatty acids (FFAs) in plasma [59], and harbours seven binding sites that are common to FFAs with a range of chain-lengths (C10-C18) across its three domains (Fig. 1a) [60,61]. *In vitro*, saturated, mono- and poly-unsaturated FFAs, with chain lengths ranging from C6 to C24, can be bound [62]. FFA affinities of albumins from different species are similar, although the effects of FFA binding on protein properties may vary, and drawing conclusions for an albumin from one particular mammalian species using data for another has been discouraged [62]. Binding constants are chain-length dependent (K_D_ ≈ 1.5–35 nM for the strongest sites), with C18:1 (oleic acid) displaying the highest affinity [62,63]. Under normal physiological conditions, oleic acid is the most abundant albumin-bound FFA *in vivo*, followed by palmitate (C16), linoleate (C18:2) and stearate (C18) [59], all of which also bind with affinities in the low to mid nanomolar range [[62], [63], [64]].

FFA-binding sites fall into at least three affinity categories [57,65]. FA2, 4 and 5 are the primary high-affinity sites, followed by the medium-strength sites FA1 and 3, and then FA6 and 7, the weakest sites that are common to most chain-lengths and detectable by X-ray crystallography [60,66] (Fig. 1a). Correlation of affinity with location was achieved by combining X-ray crystallography, site-directed mutagenesis, and ^13^C NMR spectroscopy [65]. The latter studies employed palmitate labelled with ^13^C at the carboxyl carbon, an approach that had previously been developed for a range of FFAs (C8-C18) binding to BSA [[67], [68], [69], [70]]. The ^13^C NMR methodology also allowed deducing that at least four sites on BSA involved the formation of ion-pair interactions with arginine or lysine residues, and indeed, their formation was confirmed by X-ray crystallography [60]. All three high-affinity sites are fairly enclosed and accommodate FFAs in extended conformations. The carboxylate headgroups form salt bridges with arginines or lysines, with further stabilisation from hydrogen bonds from tyrosines or serines. Sites FA1 and 3 also involve ion pairing with arginines, but are overall more accessible, and tend to bind FFAs in “curled” conformations. Sites FA6 and 7 are the most surface-exposed sites, and do not involve any ionic interactions.

Under basal physiological conditions albumin typically carries 0.1–2 molar equivalents (mol. equiv.) of FFA. When levels of FFAs are elevated, such as during intense exercise, fasting or pathological conditions such as diabetes, albumin can bind in excess of 6 mol. equiv. of FFA [59]. Interestingly, increased concentrations of non-albumin bound FFAs are known to enhance insulin secretion and reduce growth hormone levels [71]. Additionally, glucose can impair FFA binding to albumin, which has been implicated in the progression of type 2 diabetes [65,72]. It is thus clear that albumin is a critical component of energy-metabolism related networks. In 3, 4, we will outline how plasma FFA levels directly, via albumin, may affect zinc speciation and distribution. Thus, the role of albumin goes far beyond acting as a mere carrier of zinc and FFAs, but mediates their crosstalk.

## 3 Albumin enables crosstalk between zinc and fatty acids

**Fig. 2:**
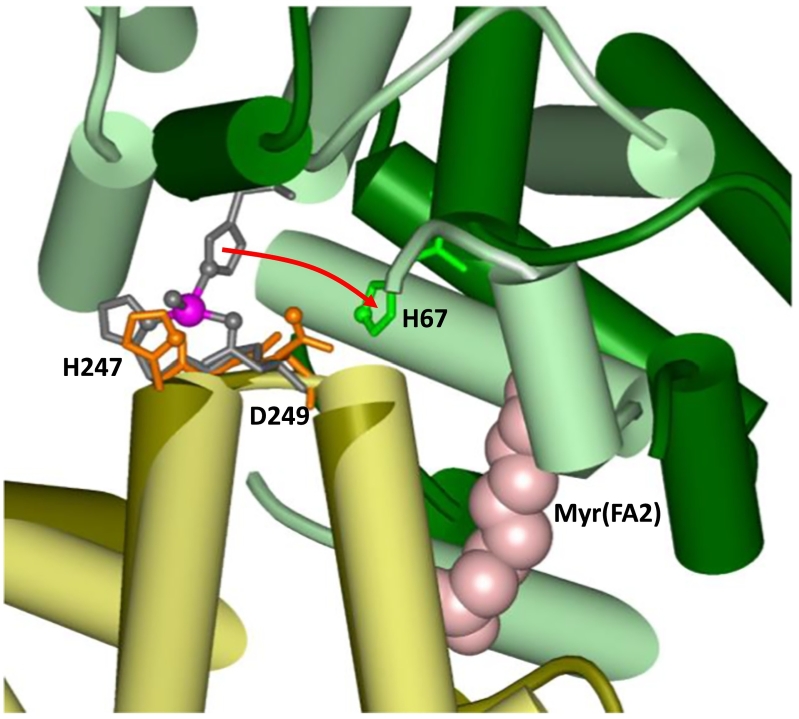
Effect of FFA-binding to site FA2 on zinc site A. The image has been generated by structural alignment of domain II of HSA in its Zn-bound form (PDB: 5IJH [33]; coloured in light yellow and light green, with zinc-binding residues in grey) with domain II of myristate-bound HSA (PDB: 1BJ5 [61]; coloured in dark yellow and dark green, with zinc-binding residues in orange and green). Myristate in site FA2 (light pink) leads to a relative movement of Nε2 of His67 by 7.5 Å (red arrow).

The binding of Zn^2+^ and FFAs to serum albumin is not independent from one another, but linked by allostery [32,55,56]. This is due to the fact that the high-affinity sites A (for Zn^2+^) and FA2 (for FFAs) both lie at the interface between domains I and II, and require interactions with residues from both domains. Crucially, Zn-site A is only available in FFA-free albumin [36], but is disrupted when an FFA molecule occupies site FA2 [32,33], because this binding event requires significant changes to the domain I/II interface. FA2 is formed by two half-pockets, one of which is located in sub-domain IA, and the other in sub-domain IIA. The carboxylate headgroup interacts with the positively charged guanidinium group of an arginine (Arg257) at the bottom of the pocket in sub-domain IIA. This electrostatic and hydrogen bonding interaction provides a significant amount of binding strength, but the domain IA sub-pocket must be lined up for complete accommodation of an FFA molecule. For zinc site A, this has the effect of a ‘spring-lock’ mechanism, in which coordination from domain I (His67) and domain II (His247 and Asp249) is disengaged, causing the release of Zn^2+^ from site A [48] (Fig. 2). This is only the case for FFAs with ten or more carbon atoms; the C8 FFA octanoate, if bound to the domain IIA sub-pocket, is too short to elicit the allosteric switch required for crosstalk between FFA and Zn^2+^ [60], although it still disturbs Cd^2+^ binding to site A, as studied by ^111^Cd-NMR [12].

**Fig. 3:**
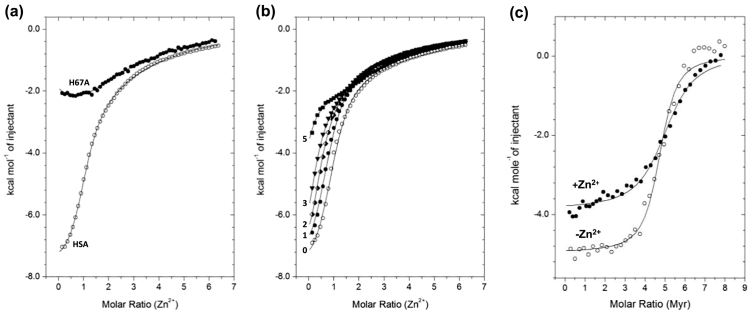
Isothermal titration calorimetry (ITC) data for the modulation of Zn^2+^ binding to albumins. (a) Relative to native HSA, mutation of H67A disrupts albumin's ability to bind Zn^2+^ by a factor of approximately 5. (b) Increasing equivalents (0–5 mol. equiv.) of myristate (C14:0 FFA) reduce the ability of HSA to bind Zn^2+^. (c) The presence of 1 mol. equiv. of bound Zn^2+^ alters the energetics but not stoichiometry of the binding reaction of myristate (Myr) to BSA. The decrease in exothermicity can be explained by the energetic cost of first breaking the Zn^2+^-albumin bonds before Myr can bind to FA2. Data in (a) and (b) were acquired at near-physiological pH and ionic strength (50 mM Tris, 140 mM NaCl, pH 7.4) [33,56]; experiments in (c) were carried out in pure water as this caused fewer problems regarding fatty acid solubility [55].

Analysis of published albumin X-ray structures had suggested that an allosteric link may exist, but could not establish whether Zn^2+^ prevented FFA binding or vice versa. This question was answered through competition experiments monitored by isothermal titration calorimetry (ITC; Fig. 3) for BSA [55], HSA, and the HSA H67A mutant [56]. ITC provides access to affinity data for both metal ions and FFAs [55,73]. In the absence of FFA, Zn^2+^ binding to all three proteins under these conditions was exothermic and could be fitted to a “two sequential sites” model (concentrations were too low to capture a third Zn site). From the fitted data (and taking into consideration competition from the Tris buffer), the *K*_d_ values for site A on HSA and BSA were 1.7 ± 0.3 μM [33] and 0.12 ± 0.08 μM [55], respectively. The corresponding *K*_d_ value for the HSA H67A mutant was 9.1 ± 2.9 μM, indicating that the H67A mutation weakened binding to HSA by a factor of approximately 5 when studied by ITC [56], providing independent confirmation of the location of site A. Similar conclusions were previously reached by equilibrium dialysis [12]. Affinities to site B were over one order of magnitude lower [33,55].

**Fig. 4:**
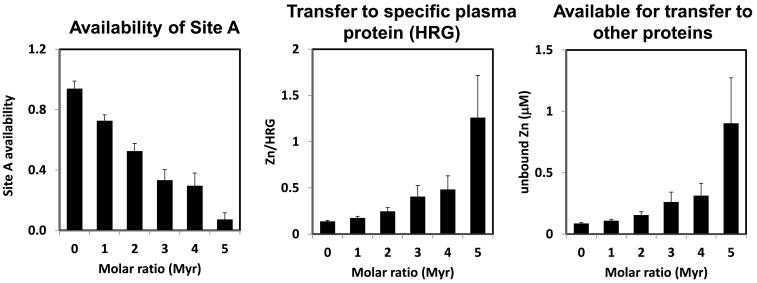
Evaluation of ITC data and plasma zinc speciation modelling for a system consisting of 620 μM HSA sites A and B, the abundant zinc-binding protein HRG at 1–2 μM, 15 μM Zn^2+^, and varying amounts of fatty acids (0–3.1 mM). The Figure is based on data published in reference [56].

The most significant results from this work concern the clearly adverse effect of the presence of FFA (myristate) on Zn^2+^ binding (Fig. 3b). Fitting of the data was accomplished by keeping the affinity constant determined for FFA-free HSA, and using the molar fraction of binding site A as a fitting parameter. This demonstrated that with increasing levels of FFA, availability/occupation of site A is progressively diminished (Fig. 4) [56]. In contrast, the stoichiometry of fatty acid binding is not affected by the presence of Zn^2+^ (Fig. 3c). Hence, and also bearing in mind that most circulating albumin molecules will, on average, have at least 1 FFA molecule bound, whilst only ca. 2% of albumin molecules will carry a Zn^2+^ ion, FFA levels drive zinc speciation, but not vice versa. Whilst FFA effects on zinc binding are most dramatic at the highest FFA loadings, it is important to realise that effects are already apparent between 0 and 2 mol. equiv. FFA, i.e. in physiologically normal ranges. The possible impact of this albumin-mediated crosstalk between two important signalling networks is outlined in the following section.

## 4 Implications of plasma zinc and fatty acid interplay

The crosstalk between FFAs and zinc may have far-reaching consequences for normal physiology and in the pathology of diseases associated with fatty acid biochemistry and zinc toxicity and/or cell signalling. Because around 98% of all mobile zinc in plasma are bound to albumin, even small changes in albumin's capacity for zinc binding may have significant consequences. The decrease in zinc binding capacity reflected in Fig. 3b will affect plasma Zn^2+^ speciation. Indeed, a simple speciation model involving ITC-derived affinity data for HSA sites A and B in the presence and absence of myristate, and a single potential zinc acceptor protein (histidine-rich glycoprotein (HRG); see further below) at physiologically relevant concentrations suggests that FFA-binding dramatically shifts the distribution of Zn^2+^ in plasma (Fig. 4). The model system is of course quite simplistic, especially regarding the presence of a single acceptor protein, which does not adequately reflect the large number of plasma and cell surface proteins with potential zinc-binding capacities. In the aforementioned model, the minimal proportion of Zn^2+^ that would be available for interaction with these other proteins is illustrated by the right-hand chart of Fig. 4.

Thus, the three major consequences of increases in plasma FFA levels that can be anticipated concern (a) zinc re-distribution to other plasma proteins with Zn-binding ability, (b) export of excess free Zn^2+^ from plasma, via interaction of free Zn^2+^ with membrane-bound ZIP transporters [32], and (c) zinc binding to cell-surface receptors and modulation of their function [9,74]. The latter two processes may occur for any cells in contact with plasma, including immune cells and endothelial cells. Zinc uptake by endothelial cells is the crucial step for re-distribution of Zn^2+^ from plasma to tissues. Accordingly, our hypothesis predicts that elevated plasma FFA might promote zinc efflux from plasma. Regarding process (c), zinc-sensing receptors are present in a variety of cell types including vascular endothelial cells [74] and neurons [9,75].

We note that zinc speciation at 0 and 2 mol. equiv. of myristate is already markedly different; this means that FFA—Zn^2+^ crosstalk is in operation under normal physiological conditions. At present, there are few data to allow tracking of the effects of such physiological crosstalk, although there are some indications that physiological states that are associated with elevated plasma FFA, such as moderate exercise, are also associated with decreased plasma levels of zinc [76]. More data are available to find correlations under extreme conditions, for instance disease states such as metabolic syndrome [77], diabetes [78,79], and cardiovascular disease [33,80] – all of which are characterised by elevated plasma FFA levels and depressed plasma zinc concentrations [81]. We suggest that albumin is at the centre of a causal relationship that links these independent observations, and that the FFA-modulated zinc speciation leads to increased zinc export from plasma into cells. In the following sections, we will consider both changes in zinc speciation in plasma, as well as potential effects of shifting zinc from plasma to cells in relation to metabolic, cardiovascular and neurodegenerative diseases, respectively. An overview of these potential effects and how they may contribute to particular disease states is outlined in Table 1.

**Table 1:** Conditions associated with changes in plasma zinc speciation, which may be impacted by changes in the levels of free fatty acids (FFAs), and observations consistent with our central hypothesis.

Condition		Observations and link to Zn/FFA crosstalk	Reference
Strenuous aerobic exercise		Enhanced export of zinc from plasma, leading to drop in plasma Zn^2+^	[82]
Obesity		"	[83]
Type 2 diabetes		"	[84]
Coronary heart disease		"	[85]
Myocardial ischemia		"	[[86], [87], [88]]
Heart failure		"	[89]
Insulin resistance/sensitisation		1. Dissociation of insulin hexamer to active monomer promoted by albumin reducing the concentration of free Zn^2+^– adversely affected by high plasma FFA. 2. Zinc binding to intracellular protein tyrosine phosphatase 1B inactivates this enzyme, leading to increased insulin receptor signalling.	[15,90,91]
Inflammation		Systemic zinc deficiency linked to chronic inflammation. High FFA known to activate inflammation pathways.	[92,93]
Thrombosis		*Re*-distribution (speciation change) of plasma zinc from albumin to histidine-rich glycoprotein (HRG) to enhance HRG-mediated heparin neutralisation.	[56]
Myocardial ischemia		‘Ischemia-modified albumin’, as determined by the albumin‑cobalt-binding assay, provides indirect measure of plasma FFAs.	[56]
Alzheimer's disease		1. Albumin is major binding partner for cytotoxic Aβ, FFA, and Zn^2+^, and FFA affect both Aβ and Zn^2+^ affinity. 2. FFAs implicated in depletion of extracellular free zinc pools, leading to lower incidence of neurodegeneration.	[94] [95]

## 5 Energy metabolism and metabolic diseases

Numerous associations between Zn^2+^ and endocrine signalling in relation to energy metabolism have previously been established [96]. Notably, Zn^2+^ affects either production, secretion, or activity of glucagon [97], insulin [98], adiponectin [99], and leptin [22]. Particularly intriguing interactions in the context of FFA-Zn crosstalk have been identified for insulin and leptin. Insulin is critical for glycemic control and stimulates glucose uptake from plasma into cells, and leptin regulates organismal energy uptake and expenditure [100].

### 5.1 Zinc and insulin

Insulin is produced in pancreatic β-cells, and stored in secretory granules that are extremely high in Zn^2+^ (10–20 mM). Accumulation of Zn^2+^ in secretory granules is mediated by the ZnT8 zinc efflux transporter protein. A common polymorphism in ZnT8 has been implicated in increased susceptibility to type 2 diabetes [15], and ZnT8 is a critical auto-antigen in type 1 diabetes [101]. Every time insulin is secreted into the extracellular matrix and the circulation [90], this coincides with the secretion of free Zn^2+^. A direct inhibitory effect of the co-secreted free Zn^2+^ on glucagon-secreting pancreatic α-cells has been suggested [102,103]. It is also noteworthy that many clinically-used insulin preparations utilise significant amounts of Zn^2+^ not only as a stabiliser, but also as an agent to modulate the pharmacokinetics of insulin [104]. The presence of Zn^2+^ stabilises the inactive hexameric form of insulin that is held together by two Zn^2+^ ions bound to the six histidine residues at position B10 [105]. Dissociation into the active monomeric form requires these coordinative bonds to be broken, with free [Zn^2+^] directly affecting the concentrations of hexameric complex and monomer. It has recently been shown that dissociation into active monomer appears to be promoted *in vivo* by HSA, which effectively acts as a ‘zinc scavenger’ protein [91], and reduces free [Zn^2+^]. This means that albumin may take an active role in the activation of insulin.

Further support for this hypothesis comes from the divergence of guinea pig insulin and albumin when compared with these proteins from other mammals. The preproinsulin sequence in guinea pigs lacks the zinc-binding His(B10) which is otherwise conserved in mammalian insulins [106]. Concomitantly, guinea pigs are the only mammal that is known not to possess site A in its albumin (His67 is substituted by an Ala residue at this position) [33]. This suggests that in guinea pigs there is no requirement for albumin to bind Zn^2+^ at site A. We hypothesise that such binding may be important for insulin de-complexation (through chelation of bound Zn^2+^) in other mammals including humans. If this is the case, it may be further predicted that this process may be inhibited by the presence of FFAs in the blood by way of the described allosteric switch between sites A/FA2 of albumin. Thus, the presence of FFAs is expected to hinder albumin's ability to promote Zn^2+^ removal from the insulin hexamer, thereby rendering secreted insulin ‘inactive’ (i.e. keeping it in its hexameric form) for longer, but also reducing insulin clearance by the liver. This hypothesis is particularly interesting as plasma FFA concentrations are higher in individuals with type 2 diabetes relative to controls [107] and have been proposed to play a role in insulin resistance [108].

Related to this suggestion, it is noteworthy that fluctuations in plasma Zn^2+^ concentrations have also been associated with insulin resistance [109], and more generally, the increased risk and onset of type 2 diabetes mellitus. Zinc status and dietary Zn^2+^ supplementation in diabetes have been extensively reviewed [110]. The reduced levels of total body and plasma zinc that are common in type 2 diabetics are proposed to be a direct consequence of hyperglycemia which decreases efficient re-absorption of Zn^2+^ in the kidneys. Conversely, zinc supplementation in prediabetes was not only found to reduce blood glucose levels, but also to reduce insulin resistance and the progression to diabetes [111], whilst contrastingly, increased intake of iron and copper were associated with a higher risk of type 2 diabetes [112]. The molecular mechanism for the “insulin-mimetic” effect of zinc is thought to involve several components of the insulin signalling pathway including extracellular signal-regulated kinase 1/2 (ERK1/2) and phosphatidylinositol 3-kinase/protein kinase B (PI3K/Akt) pathways [113]. This is likely to be, at least in part, mediated by inhibition of protein tyrosine phosphatase 1B [114,115]. PTP1B dephosphorylates and thus inactivates the insulin receptor. An increase in intracellular free Zn^2+^ inhibits PTP1B, leading to active receptors, insulin uptake and subsequent signalling cascades that ultimately promote cellular glucose uptake. The increase in intracellular free Zn^2+^ is thought to originate from intracellular stores (mainly the endoplasmic reticulum) but it remains to be seen whether sporadic increases in extracellular free Zn^2+^ might also contribute to this regulatory mechanism, and whether and how the Zn-FFA crosstalk via albumin contributes to systemic zinc dyshomeostasis in metabolic syndrome and type 2 diabetes. We also note that a very recent report has revealed that extensive glycation dramatically reduces the zinc-binding ability of albumin [116]; therefore both high FFA and high blood glucose may work together to adversely affect zinc speciation in type 2 diabetes.

### 5.2 Zinc and leptin

PTP1B also inactivates the leptin receptor, hence PTP1B inhibition by elevated intracellular free Zn^2+^ is also expected to lead to enhancement of the leptin signalling pathway. Leptin is a hormone produced by adipocytes. One target of leptin signalling is the hypothalamus, where it influences appetite, with higher leptin levels signalling satiety. The effect of zinc on leptin signalling may at least in part account for the finding that reduced appetite is a symptom of zinc deficiency [117]. Moreover, an *in vitro* study demonstrated that treatment of adipocytes with zinc significantly increased leptin production, suggestive of a direct effect of extracellular zinc on leptin synthesis [22]. It may also be significant that zinc levels in adipose tissue are inversely correlated with circulating leptin (and insulin) in rats fed a high-fat diet [93]. Furthermore, for many obese individuals, hyperleptinemia is coupled with hypozincemia (low plasma zinc) [22,118]. Indeed, correlations between either zinc deficiency or, more precisely, reduced plasma levels of zinc, and dyslipidaemia are recognised [119], but in this case, cause and consequence are not clear-cut. Overall, it appears that there are multiple, multi-directional links between zinc and lipid metabolism [15,120], and in some of these, HSA could play a direct role.

### 5.3 Zinc-a2-glycoprotein

At least one other plasma protein has been reported to bind both zinc and FFAs, and should therefore be mentioned here. The adipokine zinc-α2-glycoprotein (ZAG) stimulates fat mobilisation and lipolysis in adipose tissue [121]. The origin of the name ‘ZAG’ stems from the observation that high concentrations of zinc (>900 μM) lead to protein oligomerisation and precipitation [122]. Though none of the reported ZAG crystallographic structures display a bound zinc ion, one strong zinc binding site and up to 15 weak zinc binding sites have been identified using molecular dynamics simulations [122].

**Fig. 5:**
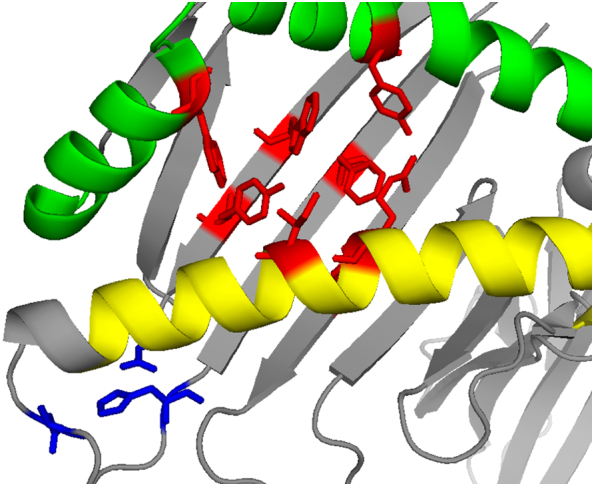
Zinc-α2-glycoprotein can bind FFAs and Zn^2+^ ions. X-ray crystallographic structure of zinc-α2-glycoprotein (ZAG) showing hydrophobic sidechains Tyr14, Arg73, Ile76, Phe101, Trp115, Tyr117, Trp148, Tyr161 (**red**) involved in the postulated inter-domain FA binding site between α1 (**yellow**) and α2 (**green**) helices (PDB: 1T7V) [123,126,127]. The high affinity zinc-binding site shown (**blue**) involving Asp90, His95 and Asp120 has been predicted using molecular modelling, and is located nearby to the FA binding site [127].

ZAG can also bind fatty acids, an observation proposed to account for the regulatory role of ZAG in lipid metabolism [123]. X-ray structures of native and recombinant ZAG reveal a hydrophobic binding pocket suitable for FFA-binding (Fig. 5) [123,124]. Interestingly, the affinity of ZAG for fatty acids appears to be reduced in the presence of excess (500–2000 μM) zinc, likely by impairing the FFA binding sites by Zn-induced oligomerisation [125]. Whilst it is unlikely that ZAG would encounter such excessive Zn^2+^ concentrations in the extracellular space, it has been speculated that ZAG‑zinc oligomers may be stored in pancreatic β-cells in an analogous fashion to insulin storage [125]. However further studies are required to establish whether the interplay between zinc, fatty acids and ZAG contribute to the formation of pancreatic amylin plaques in Type 2 diabetes [125].

Considering the large concentrations of Zn^2+^ required to affect FFA binding, and the small overall quantity of ZAG-bound FFAs, it is not envisaged that ZAG mediates any significant crosstalk between zinc and FFAs in plasma, and there is no evidence that ZAG plays any role in zinc homeostasis.

## 6 Cardiovascular disease and haemostasis

Increased plasma FFA are not only a hallmark of metabolic syndrome, dyslipidaemia and type 2 diabetes, but are also implicated in several ways in cardiomyopathy [128] and other diseases of the circulatory system. Several observations indicate their possible impact on plasma zinc speciation and export from plasma in the context of cardiovascular disease (or its diagnosis) and are described in the following sub-sections.

### 6.1 Myocardial ischemia

The concentrations of plasma FFAs have been observed to sharply increase in cases of myocardial infarction [129,130], presumably due to reduced consumption by the heart muscle. This will lead to high FFA-loading of serum albumin, and we have proposed [33,55,131] that this state of albumin corresponds to the clinically approved biomarker “ischemia-modified albumin” (IMA) [[132], [133], [134], [135]]. IMA is quantified by the albumin‑cobalt-binding (ACB) assay, by indirectly measuring the reduced cobalt-binding ability of HSA. The ACB assay is FDA-approved for the assessment of myocardial ischemia, but suffers from low diagnostic specificity [136,137]. Like other divalent cations, Co^2+^ can bind to site A, although site B has been identified as the highest-affinity site [138]. Our ITC experiments have demonstrated that cobalt-albumin binding is also affected by the presence of fatty acids (e.g. myristate) in a similar manner to Zn^2+^, and elevated ACB readings can be elicited simply by adding FFA to albumin [73]. Indeed, previous clinical work had highlighted a correlation between FFA levels and ACB results, although no causative molecular mechanism was proposed [135]. In essence, the ACB assay is best described as a measurement of the FFA/albumin ratio in serum [73]. The identification of IMA as HSA with a high FFA load explains essentially all clinical observations, including the lack of specificity: Elevated FFA levels are a hallmark of numerous other conditions and diseases including cancer [139], diabetes [78], and stroke [140]. Invariably, positive ACB assays have been found in these and many other conditions [73,131]. We also note that our hypothesis would predict depressed serum zinc levels for all conditions that elicit positive ACB assays, and this appears to be the case [131]; some of these conditions are listed in Table 1.

### 6.2 Thrombosis and haemostasis

The downstream impact of changes in FFA-induced plasma zinc speciation have also attracted interest in the context of blood coagulation [56,141]. HRG is in relatively high abundance in human plasma (1.5 μM). It has a high content of histidine and proline residues (each ca. 13% of the total amino acid composition) [142], making HRG well-adapted to bind various divalent metal ions (e.g. Co^2+^, Ni^2+^, Cu^2+^, Zn^2+^, Cd^2+^, and Hg^2+^) [32]. Human HRG can bind up to 10 mol. equiv. of Zn^2+^ [32], and so presents a likely recipient when considering zinc speciation in blood plasma after Zn^2+^ displacement from albumin by FFAs. Modelling of such changes in Zn^2+^ speciation suggested that a significant proportion of Zn^2+^ released from HSA upon FFA modulation may bind to HRG [56] (Fig. 4b). Zn^2+^ binding to HRG enhances its interaction with various other biomolecules, including heparin and heparan sulfate [143]. Increased HRG-heparin complex formation is therefore thought to have a pro-coagulatory effect [32], through neutralisation of anti-coagulant heparins. Interestingly, Zn^2+^ has also been shown to enhance heparin neutralisation through binding to fibrinogen [144]. In the same study it was shown that Zn^2+^ binds fibrinogen with a *K*_d_ of 9 μM, suggesting this interaction could similarly be promoted by FFA-mediated displacement of Zn^2+^ from HSA. Such interactions may lead to an increased risk of thrombotic disease in patients with elevated levels of FFAs, via modulation of HRG-heparin or fibrinogen-heparin interactions in a Zn^2+^-dependent manner. Zn^2+^ ions have also been shown to directly influence fibrin clot formation and lysis. For example, Zn^2+^ promotes clot stability by increasing the rate of clot formation [145], and also delays clot lysis by attenuating plasmin-mediated fibrin degradation [146]. Hence, earlier-stage monitoring of plasma FFA levels (which could involve repurposing of the ACB assay that measures ischemia-modified albumin, and is therefore representative of plasma FFA levels) may be a powerful diagnostic tool to guide interventions to prevent thrombosis [56].

Finally, it should be pointed out that both metabolic and cardiovascular disorders are also characterised by chronic low-level inflammation, which coincides with mild zinc deficiency [92,147]. There is considerable overlap between processes involved in haemostasis and inflammation. FFAs are known to activate inflammatory pathways, and it has been proposed that chronic FFA overload may cause endothelial stress and activation [148]. It is commonly suggested that the pro-inflammatory action of FFAs may involve oxidative stress, but we propose that this may also be mediated by their effects on plasma zinc speciation and/or zinc fluxes. The FFA-mediated increase in free Zn^2+^ may have relevance for the recent finding that the zinc-sensing receptor ZnR/GPR39 regulates endothelial cell activity [74]. Previously, correlations between zinc status and interleukin (including IL-1β, IL-2, IL-6) and tumour necrosis factor-α production in cardiometabolic disease have also been highlighted [92].

## 7 Zinc, albumin and FFAs in the brain: Implications for neurological disorders

Zinc is by far the most abundant d-block metal ion in the brain, is involved in learning and memory and affects mood [[149], [150], [151], [152]]. In turn, both dietary zinc deficiency and alterations in plasma/serum zinc levels are associated with neuropsychiatric disorders [153] including major depressive disorders [151,154] and neurodegenerative diseases including Alzheimer's disease (AD) [20,155,156]. Due to the complexity of the homeostatic and signalling networks involved, it is not straightforward to dissect causes and consequences in such correlations [157,158], but it is possible that low plasma zinc is a sign of systemic zinc dyshomeostasis. We are not aware of a direct correlation with plasma FFA levels in the case of neurological disorders, but it seems worthwhile to consider whether crosstalk between zinc and FFAs may also operate in other extracellular media such as cerebrospinal and interstitial fluid (CSF and ISF).

Although measurements of brain zinc compartmentalisation and speciation are challenging for a range of reasons [149,159], there are now clear indications for localised zinc dyshomeostasis in brains affected by neurological conditions [160,161]. However, the lack of sufficiently powered clinical studies precludes concluding whether or not targeting metal homeostasis in the brain is a viable therapeutic approach for neurological disorders [162]. Our incomplete understanding of molecular mechanisms means that it is not even clear in what way the respective metal ions should be manipulated, with supplementation [158,163,164], chelation and modulation [162] all being discussed. Nonetheless, the following discussion will show that extracellular zinc concentrations and speciation play a critical role in physiological and pathophysiological processes in the brain.

To understand the impact of alterations in brain zinc homeostasis, the role of Zn^2+^ as a neurotransmitter has to be appreciated. Certain regions of the brain including the neocortex and hippocampus harbour so-called “zincergic” (or “gluzinergic”) neurons, where Zn^2+^ is co-released with glutamate and used in neurotransmission [165]. The pre-synaptic vesicles of these neurons are loaded with Zn^2+^ by the zinc transporter ZnT3 [166]. Exocytosis of these vesicles leads to temporally and spatially restricted increases of free [Zn^2+^] to 300–400 μM [167]. These zinc signals are perceived by several receptors of the post-synaptic neurons, and modulate their function [9,75,159], with various downstream effects that ultimately play a role in neuroplasticity and long-term potentiation. The latter insights go some way to explain the impact of zinc on both mood-related and cognitive disorders.

Free zinc concentrations as low as 0.1 μM are toxic to some cell types including neurons [11,168]; hence, the rapid clearance of extracellular zinc ions at hundreds of μM after exocytosis is essential. Although details of mechanisms for clearance are not known, it can be anticipated that these might also involve interactions between zinc and proteins present in the extracellular matrix. Much attention has focused on metallothioneins, in particular MT3 [169]. Yet, with 3 μM, albumin is also the most abundant protein in ISF and CSF [170], and it may be inferred that the Zn:HSA complex is of major importance for zinc speciation in these fluids as well. The total Zn^2+^ concentration in CSF is 150–380 nM [171], and basal free Zn^2+^ concentration has been determined at 5–25 nM [172]. Intriguingly, there are indications that basal extracellular zinc concentrations increase with age [159,171]. A higher extracellular free zinc concentration might in itself be harmful, either through outright zinc cytotoxicity, or by interfering with correct zinc signalling for memory and long-term potentiation.

In principle, an increase in free Zn^2+^ in ISF or CSF may result from changes in total zinc, a shift from intra- to extracellular space, or its extracellular speciation, or a combination of all three possibilities. In any case, such increases may promote the development of sporadic AD in several ways [171]. The emerging role of albumin to combat at least one of the molecular mechanisms underlying AD pathology is discussed in Section 7.1. The causes for increases in basal extracellular zinc are as yet unclear; but besides the hypothesis of decreasingly efficient re-uptake of synaptically released Zn^2+^ and subsequent leakage into surrounding areas, a direct effect of poly-unsaturated fatty acids (PUFAs) has been uncovered recently, highlighted in Section 7.2.

### 7.1 Zinc and albumin effects on Aβ aggregation

Besides having several other deleterious effects [159,171], brain zinc dyshomeostasis is suggested to critically contribute to the formation of senile plaques, one of the hallmarks of AD [171]. These plaques consist of aggregated amyloid beta (Aβ) peptides, but also contain significant quantities of Zn^2+^ (besides Cu^2+^ and Fe^3+^) [173,174]. Numerous *in vitro* studies have demonstrated that the interaction of Zn^2+^ with Aβ promotes fibril and plaque formation [175,176]. Aβ plaques form predominantly in the neocortex, one of the brain regions harbouring zincergic neurons and where thus extraordinarily high fluctuations in extracellular free Zn^2+^ occur [171]. Significantly, the locations of AD plaques and ZnT3, as a marker of zincergic neurons, match closely [159]. Moreover, the quantity of Aβ deposits also correlates with X-ray-fluorescence-detected zinc in surrounding tissue [173]. It is therefore likely that free extracellular zinc directly contributes to Aβ plaque formation. Although the plaques are not the most pathogenic form of Aβ, one major question in this scenario concerns what it is that changes between a healthy young brain and an aged brain affected by sporadic AD. One hypothesis proposes that a higher basal extracellular free Zn^2+^ concentration in the aged brain is at least a contributing factor [159,171].

Whilst there is at present no indication that the albumin-mediated Zn-FFA crosstalk is in operation in the brain's extracellular media, both albumin [177,178] and FFAs [94,95] have recently been found to affect Aβ aggregation, in addition to Zn^2+^. There is some exchange between plasma and CSF across the blood-brain barrier, and as a consequence, Aβ is not only present in brain, but also circulates in plasma – overwhelmingly as complex with HSA [178,179]. Significantly, this interaction is not only critical for Aβ clearance, but also inhibits Aβ fibril formation at physiological (μM) concentrations in the brain interstitium [180]. HSA is not only able to stop fibrillisation of Aβ monomers, but more importantly, also traps and “neutralises” the oligomers that are thought to be the most cytotoxic form of Aβ [94]. HSA's critical role in Aβ sequestration and clearance has been the subject of a phase II clinical trial, which showed cognitive improvement in AD patients undergoing albumin-plasma exchange [30]. We suggest that functional HSA may influence Aβ aggregation potentially in two ways: by direct binding of the peptide, but also by reducing free [Zn^2+^]. The latter hypothesis is as yet untested, but it is intriguing that the loading of HSA with palmitate (C16:0) reduces its ability to inhibit Aβ fibre formation [94]. Even though non-FA2 binding ligands including warfarin and cholesterol also abrogated the ability of HSA to inhibit Aβ fibre formation [94], the interplay between metal ions, Aβ, HSA, FFAs and other endogenous HSA ligands clearly warrants further investigations.

### 7.2 PUFA/zinc interactions in the brain

Certain polyunsaturated fatty acids (PUFAs) are of particular interest in neurochemistry and neurodegeneration. A recent report suggested that very high concentrations of Zn^2+^ may impair the affinity of HSA for linoleic acid [181], but the Zn^2+^ concentrations employed (1 mM) were not physiological. A more clear-cut link between PUFAs and zinc in the brain, albeit without involvement of albumin, concerns omega-3 fatty acids, which are well-known to prevent age-related cognitive decline including AD [182]. Intriguingly, it has been demonstrated that their beneficial effects include acting on brain zinc homeostasis: Deficiency of docosahexaenoic acid (DHA) has been shown to increase extracellular free Zn^2+^ levels in the hippocampus [95,183]. This is thought to be mediated by affecting the expression of the zinc transporter ZnT3, which is responsible for Zn^2+^ transport from the cytoplasm of neurons into synaptic vesicles [166]. There is an inverse relationship between DHA levels and ZnT3 expression, with increased levels of DHA decreasing ZnT3 expression levels. This is expected to reduce loading of synaptic vesicles with Zn^2+^, and might, on average, reduce the concentration of free Zn^2+^ in the extracellular space. This could counter the previously mentioned increase of extracellular free Zn^2+^ in old age, and could therefore be overall neuroprotective. It is not known by which mechanism DHA affects ZnT3 expression, but neuronal zinc homeostasis may prove to be more profoundly affected by PUFAs, as a DHA-induced reduction in overall cellular Zn^2+^ influx has also been reported [184]. While the biochemistry underpinning the interaction between DHA and Zn^2+^ levels has not yet been fully elucidated, the impact of FFAs on zinc fluxes and consequences for the progression of neurodegenerative diseases calls for further exploration.

## 8 Conclusions

Although a plausible molecular mechanism and a vast array of co-incidental observations are available to propose a direct impact of plasma FFA levels on zinc speciation, zinc fluxes, and zinc signalling, the physiological consequences of this interplay have not yet been studied in a systematic manner, in part due to limited interactions between the “biometals” and “lipids” research communities.

More detailed investigations into the impact of fatty acid binding to albumin on Zn^2+^ dynamics on a cellular and organismal level may provide insight into downstream implications for health and disease, in the context of energy metabolism, the cardiovascular system, the immune system, and neurochemistry. The first step towards such new insights requires an integrated, quantitative approach that considers FFA-dependent extracellular speciation and Zn^2+^ cell uptake, and correlates these data with subsequent biochemical and cellular consequences.

## Transparency document

Transparency document Image 1
